# Matrix metalloproteinases and their inhibitors in canine mammary tumors

**DOI:** 10.1186/1746-6148-7-33

**Published:** 2011-07-04

**Authors:** Luca Aresu, Mery Giantin, Emanuela Morello, Marta Vascellari, Massimo Castagnaro, Rosa Lopparelli, Vanessa Zancanella, Anna Granato, Spiridione Garbisa, Arianna Aricò, Alice Bradaschia, Franco Mutinelli, Mauro Dacasto

**Affiliations:** 1 Dipartimento di Sanità Pubblica, Patologia Comparata e Igiene Veterinaria, Facoltà di Medicina Veterinaria, Università degli Studi di Padova, Padova, Italy; 2 Dipartimento di Patologia Animale, Facoltà di Medicina Veterinaria, Università degli Studi di Torino, Italy; 3 Histopathology Department, Istituto Zooprofilattico Sperimentale delle Venezie, Viale dell'Università 10, 35020 Legnaro (PD), Italy; 4 Dipartimento di Scienze Biomediche Sperimentali, Università di Padova, viale Colombo 3, Padova, Italy

## Abstract

**Background:**

Malignant canine mammary tumors represent 50% of all neoplasms in female dogs. Matrix metalloproteinases (MMPs) and tissue inhibitors of metalloproteinases (TIMPs) are thought to be involved in tumor progression, and they are also associated with the reactive stroma, which provides structural and vascular support for tumor growth.

**Results:**

MMP-2, MMP-9 and MT1-MMP were expressed at both the mRNA and protein levels in tumor samples. MMP-2 and MMP-9 immunohistochemical reactions were evident both in the epithelial tumor cells and in the stromal compartment to varying degrees; in particular, the intensity of the MMP-2 staining was stronger in the stromal fibroblasts close to epithelial tumor cells in simple carcinomas than in adenomas. These data were supported by gelatin-zymography; bands for the active form of MMP-2 were found in 94% of carcinoma samples, compared with 17% of benign tumor samples. The gene expression and immunohistochemical results for MT1-MMP were comparable to those for MMP-2. The immunoreactivity for MMP-13 and TIMP-2 was lower in carcinomas than in adenomas, confirming the mRNA data for MMP-13 and the other MMP inhibitors that were evaluated. The active form of MMP-9, but not the active form of MMP-2, was identified in the plasma of all of the tested dogs.

**Conclusions:**

Our findings suggest that MMP-9, MMP-2 and MT1-MMP, which are synthesized by epithelial cancer cells and cancer-associated fibroblasts, play an important role in malignant canine mammary tumors. The reduction of MMP-13 and TIMP-2 could also be a significant step in malignant transformation. MMP-2 and MT1-MMP could be further evaluated as future biomarkers for predicting the progression and prognosis of canine mammary tumors.

## Background

Mammary neoplasia is one of the most common tumors in dogs, and malignant types occur in approximately half of canine mammary tumors. Invasion and metastasis are typical features of carcinomas [1,2]. The physical process of tumor invasion involves cellular disengagement from the local microenvironment, followed by degradation of the surrounding matrix and cellular movement [3]. Invasion and metastasis of malignant tumor cells is a complex multistep process, in which the initial events are disruption of the extracellular matrix (ECM) and invasion of the basement membrane. In addition, fibroblasts in the stroma of cancerous tissue can promote the proliferation and invasion of carcinoma cells and induce angiogenesis via the secretion of several ECM molecules, proteases and cytokines [4,5]. This mechanism is highly organized and involves the selective action of a group of proteases that are active at neutral pH and can collectively degrade most, if not all, components of the ECM [6,7]. These proteases are known as matrix metalloproteinases (MMPs), and they hydrolyze the protein and proteoglycan components of the ECM. Under physiological conditions, MMPs are expressed by a variety of cells and tissues. MMPs are also involved in a number of pathological processes and are thought to be responsible for the accelerated ECM breakdown that is associated with tumor invasion and metastasis [8]. Gelatinases are a subgroup within the MMP family and include MMP-2 and MMP-9. MMPs play the same role in dogs as in humans, controlling tumor invasion and progression in different tumors [9-14].

MMP-2 and MMP-9 are secreted in an inactive form, which is called a zymogen or a pro-MMP. Several types of inhibitors, called tissue inhibitors of MMPs (TIMPs), regulate MMP activity. TIMPs also function as MMP activators [15]. To exert their inhibiting or activating functions, TIMP-1 and TIMP-2 preferentially bind to MMP-9 or MMP-2, respectively [16,17]. The unbalanced activities of MMPs and TIMPs are involved in tumor progression [18]. Evaluation of the activities of TIMP-1, TIMP-2 and TIMP-3 in canine mammary tumor samples by reverse zymography has shown that low activity can be correlated with a malignant phenotype [14].

Membrane type 1 MMP (MT1-MMP) was the first MT-MMP to be identified as a major physiological activator of pro-MMP-2 in humans [9]. Studies of canine mammary tumors suggest that pro-MMP-2 activation requires the formation of a ternary complex that consists of the C-terminal domain of pro-MMP-2, TIMP-2 and MT1-MMP [19].

A new MMP inhibitor gene, called reversion-inducing cysteine-rich-protein with Kazal-motifs or RECK, was recently identified in dogs [20]; it was reported to be an endogenous MMP inhibitor and a membrane-anchored glycoprotein that has no structural homology with TIMPs but downregulates MMP-2, MMP-9 and MT1-MMP. RECK has been implicated in tumor progression [20].

The important role of another MMP family member, MMP-13, has been demonstrated in human cancer [21]. MMP-13 promotes tumor growth and progression by mediating ECM reorganization and regulating the biological activity of cytokines in skin squamous cell carcinoma [22], melanoma [23], breast cancer and colorectal cancer [24,25]. In veterinary medicine, reports of MMP-13 expression are only available for inflammatory and degenerative diseases [26,27].

The mRNA expression of MMP-2, MMP-9, TIMP-1, TIMP-2 and TIMP-3 has been extensively studied *in vivo *and *in vitro *in various human tumors [16,28-30]. In veterinary medicine, mRNA expression of these genes has been used to study canine neoplasia [12,20] and other diseases (e.g., meningitis-arteritis, chronic valvular disease, and arthritis [31-33]), but their expression in canine mammary tumors has not been specifically documented. Thanks to the sequencing of the entire dog genome, microarray technology has been used to characterize different canine mammary cell lines, progestin-induced canine mammary hyperplasia and spontaneous mammary tumors [34-37], but to our knowledge, no targeted gene expression profiling studies are available for spontaneous canine mammary tumors.

This report is the first study to consider MMP-2, MMP-9, MMP-13, MT1-MMP and TIMP-2 expression at both the mRNA and protein levels in canine mammary tumors including adenomas and carcinomas. The enzymatic activities of MMP-2 and MMP-9 were quantified by gelatin-zymography of the same homogenized tumor tissues and plasma from selected patients. Gene expression was evaluated also for TIMP-1, TIMP-3 and RECK. Moreover, the stromal compartments in these tumors were specifically evaluated.

## Methods

### Tissue sampling

Fresh tissue samples were obtained from 35 dogs that underwent surgery for mammary neoplasia. The dogs underwent surgery due to evident disease, and the explicit consent of the owner was obtained. Excised tumor lesions were immediately divided into aliquots and stored under diverse conditions for different analytical techniques. For RNA isolation, aliquots of approximately 100 mg were immersed in RNAlater^® ^solution (Applied Biosystems, Foster City, CA) and stored at -20°C until use. For histological examination and immunohistochemistry, the tissue was formalin-fixed and paraffin-embedded. For gelatin-zymography, aliquots of up to 100 mg were frozen at -20°C until use.

### Gene expression

Total RNA was isolated from control mammary glands, which were obtained from pathogen-free adult Beagles generously provided by GlaxoSmithKline Manufacturer S.p.A. (Verona, Italy) that had been used for other experimental purposes and 35 pathological samples using TRIzol^® ^(Invitrogen, Carlsbad, CA) according to the manufacturer's instructions. The samples were purified with a classical phenol-chloroform extraction step. The total RNA concentration and quality were measured with a Nanodrop ND-1000 spectrophotometer (Nanodrop Technologies, Wilmington, DE) and by denaturing gel electrophoresis.

First-strand cDNA was synthesized from total RNA using the High Capacity cDNA Transcription Kit according to the manufacturer's protocol (Applied Biosystems, Foster City, CA).

The generated cDNA was used as the template for quantitative real-time RT-PCR (qRT-PCR) in a LightCycler 480 Instrument (Roche Diagnostics, Basel, Switzerland) using standard PCR conditions. The qRT-PCR reactions consisted of 1X LightCycler 480^® ^Probe Master (Roche Diagnostics, Basel, Switzerland), 300 or 600 nM forward and reverse primers (the primer combination and final concentrations were optimized during assay setup), 100 nM human Universal Probe Library (UPL) probe (Roche Diagnostics, Basel, Switzerland) and 5 ng cDNA. The primers and human UPL probes shown in Table 1 were designed using the UPL Assay Design Centre web service. Calibration curves using a four-fold serial dilution of a cDNA pool revealed PCR efficiencies close to two and error values less than 0.2. Canine transmembrane BAX inhibitor motif containing 4 (CGI-119) and Golgin a 1 (GOLGA1) were chosen as reference genes for the absence of pathological state-dependent differences in mRNA expression [38]. Their amplification efficiencies were approximately equal to that of the target genes; moreover, no statistically significant difference was observed in their expression profiles between healthy and pathological samples. The ΔΔCt method [39] was used for the relative quantification of mRNA. Relative quantification (RQ) values were ultimately expressed as the fold change, such as RQ sample/control group mean RQ ratio, assuming that the control mammary gland mean RQ was equal to 1. Statistical analysis of the gene expression data was performed using the Kruskal-Wallis test followed by Dunn's post test. The correlation analysis of the target gene mRNA data was performed using a Spearman nonparametric test. In both analyses, GraphPad InStat 2.01 software (San Diego, California, USA) was used, and a *p *value < 0.05 was considered significant. Finally, Grubbs' test was used to identify outliers.

**Table 1:** Primer sequences and UPL probes used for qRT-PCR amplification of selected target and reference genes.

Genes	Accession number	Primer sequence (5'-3')	UPL probe
MMP-2	[GenBank:XM_535300.2]	F: gggaccacggaagactatga R: atagtggacatggcggtctc	29

MMP-9	[GenBank:NM_001003219.1]	F: tgagaactaatctcactgacaagca R: gctcggccacttgagtgta	6

MMP-13	[GenBank:XM_536598.2]	F: ctcttcttctcgggaaacca R: gcctggggtagtctttatcca	50

MT1-MMP	[GenBank:XM_843664.1]	F: gatctgaatgggaatgacatctt R: gatggccgagggatcatt	76

TIMP-1	[GenBank:NM_001003182.1]	F: cagggcctgtacctgtgc R: cctgatgacgatttgggagt	112

TIMP-2	[GenBank:NM_001003082.1]	F: atgagatcaagcagataaagatgttc R: ggaggaaggagccgtgtag	93

TIMP-3	[GenBank:XM_538410.2]	F: tgctgacaggccgcgt R: gcagttacagcccaggtga	14

RECK	[GenBank:NM_001002985.1]	F: aaggggtgtctgtctggagat R: cccaatttgcaaccttgaac	97

CGI-119	[GenBank:XM_531662.2]	F: tctacaatctaagagagatttcagcaa R: ttcctgacaagcacaaaatcc	15

GOLGA-1	[GenBank:XM_537849.2]	F: ggtggctcaggaagttcaga R: tatacggctgctctcctggt	149

F: forward; R: reverse

### Immunohistochemistry

To analyze the expression of the MMP-2, MMP-9, MMP-13, MT1-MMP and TIMP-2 proteins by immunohistochemistry, contiguous 4 μm sections were cut from blocks of formalin-fixed, paraffin-embedded tissue, and the sections were placed on charged slides. After deparaffinization, the primary antibody incubation step for all antibodies was performed by an automated system (Ventana Medical Systems, Tucson, AZ). The pertinent antibody details are summarized in Table 2. The remainder of the staining procedure included incubation with a biotinylated anti-mouse secondary antibody, the diaminobenzidine substrate and a hematoxylin counterstain was performed using the Ventana ES automated immunohistochemistry system. Negative control slides were incubated with isotype-matched immunoglobulin in parallel with each staining batch to confirm the specificity of the antibodies. For each antibody, cases were semi-quantified for each protein-stained area. The intensity and the percentage of immunoassayed tumor cells were analyzed. Each slide was scanned with a 400 × power objective in ten fields per slide, and the fields were selected by searching for protein-stained areas. A section was considered negative or positive according to the absence or presence of cytoplasmic staining. An intensity score of zero was given if no staining was detected, one if there was weak to moderate staining, two if moderate to strong staining was present, and three if strong staining was detected. A total score for each examined field was obtained by multiplying the intensity score by the percentage of immunoassayed cells. A final ratio was obtained after averaging the ten selected fields. An image analysis system that consisted of an Olympus BX51 microscope and software analysis (analySIS, Soft imaging system, Münster, Germany) was used. Furthermore, the immunoreactivity of each antibody was separately recorded for tumor and stromal cells in ten fields (400× power objective), and the final result was expressed as the average percentage from ten fields. For statistical analysis, dog tumors were subdivided into two groups: benign and malignant. Immunostaining score values for each protein were expressed as a median (range). The Mann-Whitney test was used to compare the immunostaining scores. Differences in percentages were calculated with the Chi-square test. The program SPSS 17.00 (SPSS Inc, Chicago, IL, USA) was used for all calculations.

**Table 2:** Antibody details.

Antigen	Source	Clone	Dilution	Manufacturer
MMP-9	Human	MAB 3309	1:1000	Chemicon (Millipore)

MMP-2	Human	Ab-7	1:400	Neomarkers, Fremont, USA

TIMP-2	Human	MAB 3317	1:1000	Chemicon (Millipore)

MMP-13	Human	VIIIA2	1:100	Millipore Co., Billerica, USA

MT1-MMP	Human	-	1:200	Millipore Co., Billerica, USA

### Zymography

MMP-2 and MMP-9 activity was studied by zymography, which reveals the gelatinase activity of latent proenzymes (zymogens) and mature MMPs. The homogenized tissue was centrifuged at 1500 rpm for 10 min, and the protein concentration of the supernatant was measured. The sample protein concentration was adjusted to 1 mg/ml, and 5 μl was diluted 1:1 in sample buffer; the final 10 μl sample was subjected to electrophoresis on an 8% SDS-PAGE gel copolymerized with 0.1% gelatin. Following electrophoresis, the gel was incubated for 1 h at room temperature in a 2.5% Triton X-100 solution and then at 37°C for 16 h in 0.5 M Tris-HCl buffer, pH 7.4, with 10 mM CaCl_2_. The gels were stained with 0.1% Coomassie Brilliant Blue R-250 and de-stained with 30% methanol and 10% acetic acid. Gelatinolytic activities were detected as unstained bands against the background of Coomassie-stained gelatin. Culture medium conditioned by A2058 melanoma cells was used as a control to identify the pro-MMP-9 gelatinolytic band, while conditioned media from HT1080 fibrosarcoma cells was used for the active forms of MMP-2 and MMP-9 and small amounts of the pro-MMP-2 [6]. The amount of MMP-9 in 20 μl of the A2058 melanoma cell-conditioned media was defined as 100 arbitrary units (a.u.). The MMP-2 activity in 20 μl of HT1080 fibrosarcoma cell-conditioned media was defined as 100 a.u. The bands were quantified using an image analyzer system that consisted of a GelDoc 2000 and Quantity One software (BioRad, Hercules, CA, USA).

Peripheral blood samples were collected from 14 dogs 24 h before surgery. The plasma was obtained by blood sample centrifugation and stored at -20°C, and 10 μl was used for analysis. The plasma concentrations of MMP-2 and MMP-9 were evaluated by gelatin-zymography. The gelatinolytic activity was defined as the arbitrary optical density value of the sample relative to the optical density value of the control plasma from healthy dogs.

## Results

This study included 35 dogs with a median age of 9.9 years. Of the 35 tumors, 13 were simple adenomas, and 22 were simple carcinomas, according to the WHO classification system for canine mammary tumors [40].

### Gene expression

The mRNA expression of MMP-2, MMP-9, MMP-13, MT1-MMP, TIMP-1, TIMP-2, TIMP-3 and RECK in mammary tumors and healthy mammary glands was investigated using a qRT-PCR approach. The overall results are reported in Table 3. All of the target genes were expressed in the control and tumor samples, with the exception of MMP-13, which was weakly expressed or not amplified in approximately half of the tumor samples (16 of 35 dogs). No statistically significant differences were observed for MMPs and inhibitors among the studied groups, particularly between benign and malignant tumors. Spearman correlation analysis was performed to identify potential relationships between MMPs and their preferential inhibitors at the transcriptional level and between MMP-2 and its well-known specific activator (MT1-MMP). Positive correlations were observed between MMP-2 and MT1-MMP (r = +0.52, *p *< 0.01), MMP-2 and TIMP-2 (r = +0.47, *p *< 0.01), MT1-MMP and TIMP-2 (r = +0.39, *p *< 0.05), and MMP-9 and TIMP-1 (r = +0.56, *p *< 0.001).

**Table 3:** mRNA expression of MMPs and inhibitors in control mammary glands and benign and malignant mammary tumors.

Gene	mRNA expression -**fold changes (arbitrary units)**		
	
	Control mammary gland	Benign tumors	Malignant tumors
**MMP-2**	1.00 ± 0.46	1.01 ± 0.18	0.76 ± 0.12

**MMP-9**	1.00 ± 0.30	9.95 ± 4.40	7.23 ± 1.58

**MMP-13**	1.00 ± 0.28	2.08 ± 0.70	1.27 ± 0.25

**MT1-MMP**	1.00 ± 0.24	13.26 ± 6.09	2.82 ± 0.64

**TIMP-1**	1.00 ± 0.17	4.96 ± 1.27	3.97 ± 0.66

**TIMP-2**	1.00 ± 0.34	0.49 ± 0.09	0.60 ± 0.09

**TIMP-3**	1.00 ± 0.35	4.16 ± 1.62	2.38 ± 0.65

**RECK**	1.00 ± 0.52	3.41 ± 1.77	0.51 ± 0.10

Data are expressed as the mean ± SEM.

### Immunohistochemistry

**Figure 1:**
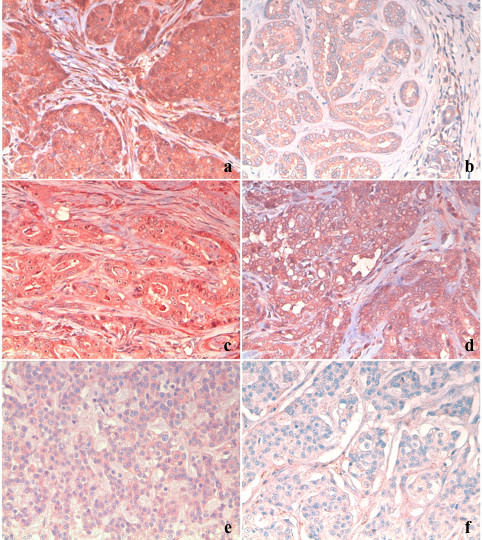
**Canine mammary tumors**. (a) Epithelial tumor cells and stromal fibroblasts with intense MMP-2 immunopositivity in a carcinoma. (b) Epithelial tumor cells with weak MMP-2 immunopositivity in an adenoma. (c) Epithelial tumor cells and fibroblasts strongly MMP-9-immunolabelled in a carcinoma. (d) Epithelial tumor cells and stromal fibroblasts with intense MT1-MMP immunopositivity in a carcinoma. (e) Moderate TIMP-2 immunostaining in epithelial tumor cells in an adenoma. (f) TIMP-2 antibody-negative epithelial tumor cells in a carcinoma. (immunohistochemistry, 200×).

Immunohistochemical staining revealed that MMP-2 and MMP-9 were present in all of the tumors examined. In all samples, the two MMPs were strongly localized in the cytoplasm of the tumor epithelial cells (Figure 1a, b, c). There were significant differences in the immunohistochemical score values for these two markers in benign and malignant neoplasias (Table 4). An intense immunoreaction was especially evident for MMP-2 in carcinomas, while the difference in MMP-9 immunolabelling in adenomas and carcinomas was lower. The MT1-MMP protein was detected in 21 tumors, and its expression was higher in malignant tumors. Tissue leukocytes and plasma cells stained positive for MMP-2 and MMP-9 as well as for MT1-MMP, which was considered a positive control. Immunohistochemical staining revealed that TIMP-2 and MMP-13 were more highly expressed in simple adenomas than in simple carcinomas (Figure 1e, f).

**Table 4:** Immunostaining score values for MMPs and TIMP-2 in benign and malignant mammary tumors, expressed as the median (range).

Protein	Immunostaining score values (median and range)		p values
		
	Benign tumors	Malignant tumors	
**MMP-2**	58.5 (45.4-145.5)	179.4 (120.1-267.3)	0.002

**MMP-9**	78 (26.8-189)	101.1 (34.3-213.9)	0.01

**MMP-13**	84.2 (0-198.2)	45.3 (0-101.2)	0.005

**MT1-MMP**	65.3 (0-230.2)	132.1 (34.2-221.2)	0.002

**TIMP-2**	74.3 (21.2-201.2)	48.9 (0-136.6)	n.s.

n.s. = not significant

The magnitude of the immunohistochemical detection of MMP-2, MMP-9 and MT1-MMP varied in the fibroblasts of the stromal compartment. The staining intensity for MMP-2 and MT1-MMP (Figure 1d) was stronger in the fibroblasts closest to the epithelial tumor cells in the malignant tumors, while the fibroblasts that were immunolabelled by the antibodies for the two proteins were scattered in the adenomas (Table 5). The percentage of fibroblasts that were positive for MMP-9 staining was lower than the percentage that were positive for MMP-2 in carcinomas. The percentage of TIMP-2- and MMP-13-positive fibroblasts was higher in adenomas than in carcinomas. The immunohistochemical data are summarized in Tables 4 and 5.

**Table 5:** Immunohistochemical score for epithelial tumor cells and fibroblasts in benign and malignant mammary tumors, expressed as the average percentage from ten fields.

Protein	Cell type	Immunohistochemical score (%)		p value
			
		Benign tumors	Malignant tumors	
**MMP-2**	Tumor cells	88.6	67.5	0.05
		
	Fibroblasts	11.4	32.5	

**MMP-9**	Tumor cells	84.1	89.4	0.002
		
	Fibroblasts	15.9	10.6	

**MMP-13**	Tumor cells	56.4	65.9	n.s.
		
	Fibroblasts	43.6	34.1	

**MT1-MMP**	Tumor cells	80.3	55.8	0.001
		
	Fibroblasts	19.7	44.2	

**TIMP-2**	Tumor cells	53.3	79.6	n.s.
		
	Fibroblasts	46.7	20.4	

n.s. = not significant

### Zymography

**Figure 2:**
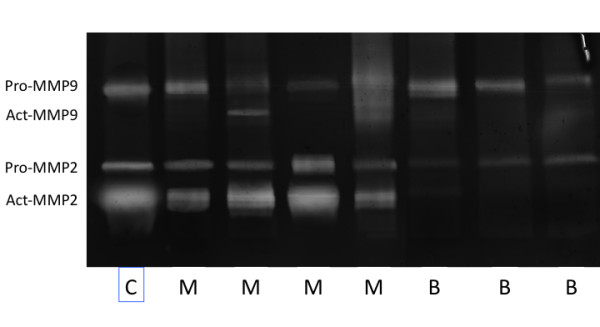
**Zymographic assay of gelatinase activity in malignant (M) mammary neoplasia, in benign (B) mammary neoplasia and in the control (C)**. Bands corresponding to latent and active forms of MMP-9 and to latent and active forms of MMP-2 were observed in carcinomas (M), while bands corresponding to latent forms of MMP-9 and MMP-2 were observed in adenomas (B). HT1080 fibrosarcoma cells (C) were used as the control for pro-MMP-9, pro-MMP-2 and active form of MMP-2.

For each tumor sample, the active and inactive forms of MMP2 and MMP-9 were measured by their gelatinolytic activities (Figure 2). The activities of MMP-2 and MMP-9 were calculated in arbitrary units per 10 ng of protein. The pro-MMP-2 band was detected in all samples examined (adenoma and carcinoma). The activity of the carcinoma samples ranged between 16.7 and 59.9 units, while the activity of the adenoma samples ranged between 3.5 and 15.7 units. Bands for the active form of MMP-2 were found in 94% of the carcinoma samples and 17% of the benign tumor samples. In the simple carcinomas, the MMP-2 activity ranged between 47.4 and 87.5 units. The pro-MMP-9 band was expressed in all samples examined by gelatin-zymography. The activity range of the carcinoma samples varied greatly (from 7.5 to 106.4 units), while the activity range of the twelve benign samples was between 20.5 and 41.2 units. Only eight carcinomas exhibited bands for the active form of MMP-9. The activity was similar among the samples, ranging from 34.3 to 40.3 units. No bands were observed for the active form of MMP-9 in adenomas.

**Figure 3:**
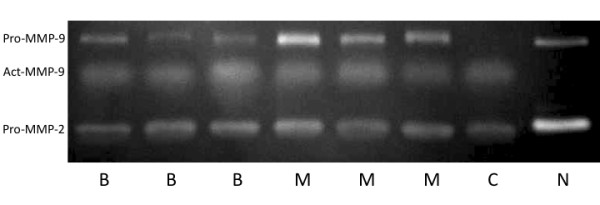
**Zymographic assay of gelatinase activity in pre-surgery plasma samples**. (M = malignant mammary neoplasia, B = benign mammary neoplasia, C = control, N = normal dog). Bands corresponding to pro-MMP-2, pro-MMP-9 and active form of MMP-9 were observed in dogs with adenomas and carcinomas (B, M). Bands corresponding to pro-MMP-9 and pro-MMP-2 were observed in the control dog, and A2058 melanoma cells (C) were used as the control for active MMP-9.

The activities of both the pro- and activated forms of MMP-2 and MMP-9 were measured by gelatin-zymography of the plasma of fourteen dogs with mammary tumors and three healthy control dogs. A representative gel is shown in Figure 3. Regardless of the tumor type, the band for pro-MMP-9 was significantly more evident in the plasma of dogs with tumors than in healthy dogs. The activated form of MMP-9 was not observed in healthy dogs but was present in all of the dogs with tumors. Pro-MMP-2 was detectable in the plasma of all dogs with tumors and also in control dogs with no difference in the level of expression, and the MMP-2 activated form was not detectable in either case.

## Discussion

In recent years, investigations of tumor growth have focused on the surrounding stroma in different tumors, which is important for cellular invasion, tumor growth and the epithelial mesenchymal transformation [41,42]. The engagement of the neoplasia with a 'reactive stroma' provides structural and vascular support for tumor growth and also leads to tissue reorganization and invasiveness [3]. MMPs play an important role in this mechanism by degrading the stromal connective tissue and basement membrane components. In addition, MMPs impact tumor cell behavior *in vivo *because of their ability to cleave growth factors, cell adhesion molecules, and chemokines. The proteolytic activity of MMPs can be regulated at different levels, including gene expression, the conversion of the zymogen to the active enzyme, and by the presence of specific inhibitors. Among MMPs, MMP-2 and MMP-9 have mainly been associated with malignant tumor progression and metastasis in both human and canine tumors [12,19,43]. In this study, the mRNA and protein levels of MMP-2, MMP-9, MMP-13, MT1-MMP and TIMP-2 in benign and malignant mammary tumors were thoroughly analyzed. We then analyzed TIMP-1, TIMP-3 and RECK mRNA expression and focused on the expression of different MMP proteins in stromal fibroblasts to identify a possible role in canine mammary tumors.

MMP-2 mRNA was expressed in all pathological samples, but no statistically significant differences were observed between benign and malignant tumors. On the contrary, at the protein level, immunohistochemistry identified higher MMP-2 expression in carcinomas; gelatin-zymography confirmed differences in MMP-2 production between adenomas and carcinomas (*p *< 0.05, data not shown). In particular, significant bands were observed for both the inactive and active forms of MMP-2 in simple carcinomas, while simple adenomas were characterized by minimal bands that corresponded to pro-MMP-2, suggesting that this enzyme is important during the infiltration process. Pro-MMP-2 may be converted to the active form during the infiltration process in malignancy; intense gelatinolytic activity has been described as necessary for malignant transformation [44]. These data are comparable to those from human studies. In human lung and gastric carcinomas, the MMP-2 activation ratio is enhanced; in canine tumor studies, higher MMP-2 and MMP-9 levels were observed in tumors than in inflammation, in malignant tumors than in benign tumors, in sarcomas than in carcinomas and in the advancing edge of canine malignances than in the center of canine tumors [11]. In this study a band corresponding to active MMP-2 was observed in only two adenomas, and histological examination of these two samples showed an intense stromal reaction close to the tumor.

Activation of the zymogen form of MMP-2 is a cell-surface event that is mediated by members of the membrane-type subfamily of MMPs, and MT1-MMP is the first physiological activator of pro-MMP-2 [45]. In this study, the immunohistochemical data for MMP-2 and MT1-MMP were comparable for both adenomas and carcinomas. MT1-MMP was distributed similarly to MMP-2 in malignant tumors and was reduced in benign tumors. Despite the opposing results obtained for mRNA and protein expression in benign and malignant mammary tumors, a close relationship between MMP-2 and MT1-MMP was observed at the pre-transcriptional level. Interestingly, immunohistochemistry showed an intense reaction for the stromal fibroblast component, with a significant increase in MMP-2 (*p *= 0.05) and MT1-MMP (*p *= 0.001) expression in the fibroblasts associated with carcinoma. MT1-MMP expression generally correlates well with MMP-2 activation in various human cancers, suggesting that MT1-MMP plays an important role in cancer cell invasion [46,47]. This presumably occurs through direct ECM cleavage by MT1-MMP and perhaps via MT1-MMP-mediated pro-MMP-2 activation. Both MMP-2 and MT1-MMP have previously been detected by immunohistochemistry in canine mammary carcinomas [19]. Our results were similar, suggesting that peritumor stromal cells may be a possible source of MMP-2 and MT1-MMP and promoters of invasion. Further studies in different tumors or *in vitro *are needed to support this hypothesis.

qRT-PCR analysis revealed a high level of mRNA expression for MMP-9 in both benign and malignant mammary tumors. Epithelial cell immunoreactivity with the anti-MMP-9 antibody was observed in all tumors. MMP-9 positivity was higher in malignant tumors than in adenomas, confirming previous observations in human breast cancer [10] and canine mammary tumors [13]. Immunohistochemistry also revealed that the stromal fibroblasts had a minimal capacity to synthesize MMP-9. The enzyme activity was analyzed by gelatin-zymography; active MMP-9 form was not observed in any of the simple adenomas, and only eight carcinomas had a band for the active MMP-9 form, while all of the examined tumors had variable pro-MMP-9 gelatinolytic activity (*p *< 0.01 data not shown). The limited presence of the active form of MMP-9 may indicate a minor role for this gelatinase within the tumor, and it appears to be of little prognostic or pathogenetic value, as observed in canine tumors [11].

TIMP-2 plays a double role, as both inhibitor and activator of MMP-2. Surprisingly, high TIMP-1 and TIMP-2 mRNA levels can predict adverse prognosis and be correlated with tumor aggressiveness in several different human cancers, including breast cancer [43,48]. In our study, neither the mRNA nor the protein levels of TIMP-2 expression correlated with malignancy.

MMP-13 was also evaluated in our study. At the pre-transcriptional level, it was expressed only in half of pathological samples, and immunohistochemistry revealed a higher level of expression in benign tumors than in malignant tumors. MMP-13 expression has previously been evaluated only in human tumors, for which MMP-13 involvement in tumor invasiveness was unclear [21].

MMP-2 and MMP-9 plasma levels have been reported to be elevated in patients with various types of cancer. In our study, plasma gelatinolytic activity in dogs was estimated by gelatin zymography. Pro-MMP-9 and pro-MMP-2 were significantly concentrated in the plasma of all of the dogs, and bands for the latent forms were present in both animals with adenoma and animals with carcinoma. Moreover, the active form of MMP-9 was present in the plasma of all of the dogs with tumors with no differences in concentration between benign and malignant neoplasias. These data could indicate that the plasma level of active MMP-9 is consistent with a proteinase activity that is due to the presence of an "ongoing disease," and MMP-9 plasma levels may be a feasible method for detecting neoplastic growth in dogs in the future.

TIMP-1, TIMP-3 and RECK mRNA expression were also evaluated and no statistically significant differences were observed among the groups. Contrasting results are reported in literature: microarray studies in dogs have shown that TIMP-1 and TIMP-3 are inhibited in progestin-induced canine hyperplasia relative to normal mammary glands [37]. Besides, a qRT-PCR study of RECK mRNA expression in various spontaneously developing canine tumors showed that expression levels were low in the majority of tumor tissues relative to normal tissues; however, in some neoplasias, RECK expression was higher than in the controls [47].

To exert its MMP inhibitory or activating role, TIMP-1 binds preferentially to MMP-9, and TIMP-2 binds to MMP-2 [16,17]. This relationship was confirmed in this study by the statistically significant correlations that were found between MMP-2 and TIMP-2, MMP-9 and TIMP-1 and MT1-MMP and TIMP-2 mRNA.

## Conclusion

The observed discrepancy between the mRNA and protein data has also been described by other studies [49]. Enzyme accumulation that is not accompanied by an increase in mRNA could be due to a feedback mechanism that shuts off mRNA expression after the secretion and/or binding of the protein. In addition, the samples used may not be representative [49]. Extracting and amplifying mRNA from cells obtained through the laser capture microdissection of the same formalin-fixed tissue that was used for immunohistochemistry might better clarify the mRNA/protein expression discrepancy observed in our study.

In conclusion, we have evaluated the involvement of MMP-2, MT1-MMP, MMP-9 and various TIMPs in canine mammary tumors, with an emphasis on the stromal compartment. The present work opens the possibility of developing new therapies and supports the use of the dog as an animal model in this field for studying the role of MMP-2 and MT1-MMP in the mechanism of cancer. MMPs may be target molecules of the switch mechanism that leads to the progression of carcinomas from adenomas.

## Abbreviations

ECM: extracellular matrix; MMP: matrix metalloproteinases; MT1-MMP: membrane type 1 matrix metalloproteinase; TIMP: tissue inhibitors of matrix metalloproteinase; qRT-PCR: quantitative real-time RT-PCR; UPL: Universal Probe Library; CGI-119: Transmembrane BAX inhibitor motif containing 4; GOLGA1: Golgin a 1; RECK: reversion-inducing cysteine-rich-protein with Kazal-motifs.

## Competing interests

The authors declare that they have no competing interests.

## Authors' contributions

LA conceived of the study, participated in its design and coordination, performed the statistical analysis and drafted the manuscript. MG designed and performed the gene expression experiments, performed the statistical analysis and helped to draft the manuscript. RML and VZ participated in the qRT-PCR design and performance. AA, SG and AB participated in the immunohistochemistry and zymography studies. EMM, AG, MV, MC and FM provided the tumor and control samples. MD participated in the design and coordination of the study and helped to draft the manuscript. All authors read and approved the final manuscript.
